# Exploring trends of running performance during matches of professional soccer players in Montenegro: A longitudinal study

**DOI:** 10.3389/fpubh.2022.966578

**Published:** 2022-08-01

**Authors:** Kosta Goranović, Rašid Hadžić, Jovica Petković, Marko Joksimović

**Affiliations:** ^1^Department of Physical Education, Faculty of Sports and Physical Education, University of Montenegro, Podgorica, Montenegro; ^2^Institute of Sports and Sports Medicine, Podgorica, Montenegro

**Keywords:** performance analysis, external monitoring, time-motion analysis, high intensity running, soccer

## Abstract

The practical value of monitoring is that well-chosen performance indicators can help coaches identify the good and bad performance of individuals or teams. External monitoring of matches is useful in establishing the physiological requirements of the sport and assessing how a player compares to the requirements of the event in this regard. This study aimed to analyze the trend component of running performance during a game of professional soccer in Montenegro. The research included a sample of 82 professional soccer players. The first subsample included 44 professional soccer players of the club Budućnost from Podgorica, height 185.89 ± 6.29 cm, mass 81.06 ± 5.47 kg, BMI 23.47 ± 0.96 kg/m^2^, age 28.86 ± 3.85 yrs. The second subsample included 38 professional soccer players from the Sutjeska club from Nikšić, height 181.88 ± 6.35 cm, mass 77.28 ± 6.78 kg, BMI 23.32 ± 1.08 kg/m^2^, age 29.43 ± 5.68 yrs. The InStat kinematic system captured the outfield players by using six cameras placed around the perimeter of the field at the minimal height of 12 m. The frame frequency was 25 frames per second; data were centralized for further analysis. Statistically significant differences were noted only in the variable sprint distance in the 2017 season. The results of the current research indicate that the soccer players who compete in Montenegro are below the values achieved by those who compete in Europe.

## Introduction

Soccer is one of the most complex sports in the world; players need technical, tactical, and physical skills to achieve successful performance and eventually win a game. The cooperative relationships between players who play different positions are critical to a team's success. For instance, the main role of midfielders is to organize the offense with proper ball control and passes, while the main duties of defenders are to win aerial duels and tackles or to perform interceptions of the balls passed to attackers. Understanding these position-specific demands is crucial in the evaluation of players' achievements (1). Modern soccer requires a high level of endurance, speed, strength, and coordination (2). Therefore, players must have well-developed physical fitness. Given that the energy used by soccer players is mainly produced by aerobic metabolism (3, 4), it is essential that players have well-developed aerobic fitness.

Running in-game performance is a set of variables used in soccer performance analysis and is defined “as the choice and combination of variables that define an aspect of performance and help achieve sporting success” (5), in which the player's duties are passing, shooting, throwing the ball, dribbling, etc. Currently, several video-based platforms are available to track player performance indicators; some of the most commonly used platforms are InStat, Optasport, and Wyscout. Such platforms quickly and accurately provide a wide range of data on game performance indicators, enabling simultaneous analysis of physical effort, movement patterns, and technical actions of players, with and without the ball (6).

Various studies have examined these characteristics and requirements within a soccer team (7). Yi et al. (8) explored the technical requirements of different playing positions for play in the UEFA Champions League. In contrast, Modrić et al. (6) identified running performance specific to each playing position in professional soccer players. Dellal et al. (9) identified positional requirements from technical and physical aspects in the French premier league. All studies indicated high applicability of running performance in evaluating team-specific achievements and team position. It is known that running performance during the game is an essential determinant of success in professional soccer, which has been studied repeatedly, although some studies have been done with different aims (10–12). However, due to its importance, more research is required in different countries according to different levels of players and leagues. This is the first study to monitor the performance of running during the game in the first Telekom Montenegrin league. In this study, we hypothesized that examining the differences in the variables mentioned in different matches could provide a useful, practical report to coaches and trainers in Montenegro. Therefore, this study aimed to explore trends of running performance during the match in professional soccer players in Montenegro in three competitive matches of different seasons.

## Materials and Methods

### Participants

The research included a sample of 82 professional soccer players. The first subsample included 44 professional soccer players of the soccer club Budućnost from Podgorica, height 185.89 ± 6.29 cm, mass 81.06 ± 5.47 kg, BMI 23.47 ± 0.96 kg/m^2^, age 28.86 ± 3.85 yrs. The second subsample included 38 professional soccer players from the Sutjeska soccer club from Nikšić, height 181.88 ± 6.35 cm, mass 77.28 ± 6.78 kg, BMI 23.32 ± 1.08 kg/m^2^, age 29.43 ± 5.68 yrs. All soccer players compete in the first Telekom Montenegrin league, the highest competitive rank in Montenegro. The study is longitudinal in nature, and testing was done in three seasons: 2014/2015, 2016/2017, and 2019/2020, where derby matches between Budućnost and Sutjeska were observed each season. The criteria for inclusion were that the first team's players had been team members for at least 6 months, that all the players went through the preparation period with the team, were without injuries in the previous 6 months, and that they played one half-season before testing. Exclusion criteria were athletes in the recovery phase from some form of acute or chronic injury and athletes who did not complete the entire preparation period. All respondents were first informed about the study and the purpose and goal of the research; the possible consequences were explained to them. Also, the procedure and the course of the testing itself were explained to the respondents. Prior to the survey, each respondent signed a consent form to participate. For this research, the consent and approval of the head coach and the club president were obtained, and testing was started. The research was in accordance with the Declaration of Helsinki (13).

### Study design

InStat Kinematic System—“Currently, various video-based systems track performance indicators of soccer players (InStat, Optasport, Wyscout). Such platforms quickly and accurately provide a large range of match-related performance measures, allowing the simultaneous analysis of the physical efforts, movement patterns, and technical actions of players, both with and without the ball” (6). “The match performance indicators for each player were determined by the position-specific InStat system. The InStat tracking system was previously employed to analyze the association between running performance and game performance indicators in professional soccer players” (6). “The InStat kinematic system captured the outfield players using six cameras placed around the perimeter of the field at the minimal height of 12 m. The frame frequency was 25 frames per second; data were centralized for further analysis. InStat Autocrop allows filming matches without a cameraman. The footage covered every player on the field. There is minimum human involvement in the process; a person is only needed to set up a panoramic camera at the required height, connect it to a computer, and check the Internet connection before the start of the match. An Autocrop camera is set at a height of 8–10 meters and 23–24 meters away from the sideline. A special algorithm allows the camera to cover the entire field. The program analyzes every frame and centers the image depending on the players' positions, without any sudden zooming. The following parameters of running performance were selected to estimate the match performance of players: total distance covered per match and during each half (m), the average speed per match and during each half (km/h), maximal speed (km/h); the total distance covered at high-intensity (m) (speed range 19.8–25.2 km/h) per match and for each half, the total distance covered sprinting (m) (speed above 25.2 km/h) per match and for each half, and the number of sprints. The speed thresholds for each category are similar to those reported previously” (6) and have been universally accepted.

### Statistical analysis

All data collected by the survey were processed using descriptive and comparative statistics. Regarding descriptive statistics, mean and standard deviation were measured for each variable. Regarding comparative statistics, a discriminant parametric procedure was used: analysis of variance with one-factor *Anova* and *Post Hoc*, which determined the differences in running performance every year separately. The statistical program for personal computers SPSS for Windows version 20.0 was used for data processing.

## Results

Table 1 shows the basic central and dispersion data on running performance during in-game soccer players. Analyzing the results in Table 1, it is evident that the players of both clubs achieved identical results in running performance during the game. Analyzing the derby match from 2020, it is evident that the soccer players of Buducnost ran more (9,129 m) in relation to the players of Sutjeska (7,019 m). Comparing the derbies from 2015 and 2017, it is clear that in the previous two derbies, the players from Sutjeska ran a greater distance compared to the derby from 2020, while the players from Buducnost ran the most in the derby in 2020. Also, in the derby in 2017, the players of both teams achieved a higher number of sprints compared to the derbies in 2015 and 2020. Applying appropriate statistical procedures, it was found that there are no statistically significant differences in running performance.

**Table 1:** Descriptive data of performance running.

**Variables**	**Team**	**2015**	**2017**	**2020**	**F**	**Sig**.
		**Mean ±SD**	**Mean ±SD**	**Mean ±SD**		
TD (m)	Budućnost	8.274 ± 3.87	8.041 ± 3.40	9.129 ± 2.46	0.760	0.541
	Sutjeska	9.441 ± 3.11	7.081 ± 3.12	7.019 ± 3.45		
WD (m)	Budućnost	2.776 ± 1.23	2.899 ± 1.23	3.431 ± 0.85	0.004	0.996
	Sutjeska	3.194 ± 0.99	2.995 ± 1.04	2.498 ± 1.28		
JD (m)	Budućnost	3.436 ± 1.67	3.175 ± 1.39	3.589 ± 1.15	1.168	0.422
	Sutjeska	3.841 ± 1.46	3.070 ± 1.37	2.795 ± 1.50		
RD (m)	Budućnost	1.378 ± 0.71	1.281 ± 0.72	1.395 ± 0.53	1.585	0.339
	Sutjeska	1.552 ± 0.72	1.283 ± 0.63	1.155 ± 0.64		
HSRD(m)	Budućnost	719 ± 0.44	583 ± 0.30	617 ± 0.28	5.389	0.102
	Sutjeska	794 ± 0.30	538 ± 0.23	461 ± 0.26		
SD (m)	Budućnost	92.75 ± 93.2	437 ± 0.32†	119 ± 0.09	0.401	0.010
	Sutjeska	105 ± 72.1	347 ± 0.23‡	66 ± 0.05		

† *2017 vs. 2015, 2020*;

‡ *2017 vs. 2015, 2020; TD, total distance; WD, walk distance; JD, jog distance; RD, run distance; HSRD, high speed runs distance; SD, sprint distance*.

**Figure 1:**
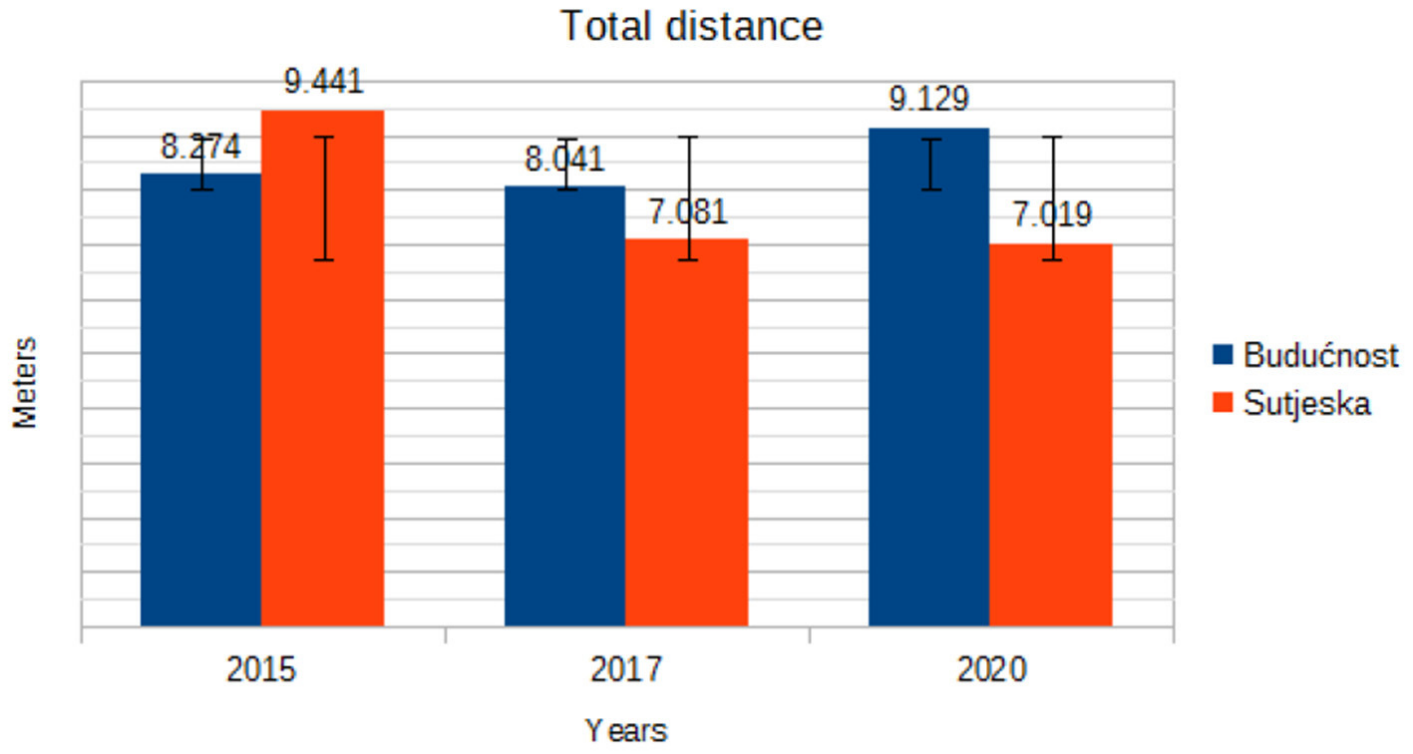
Trend in mean total distance by years.

**Figure 2:**
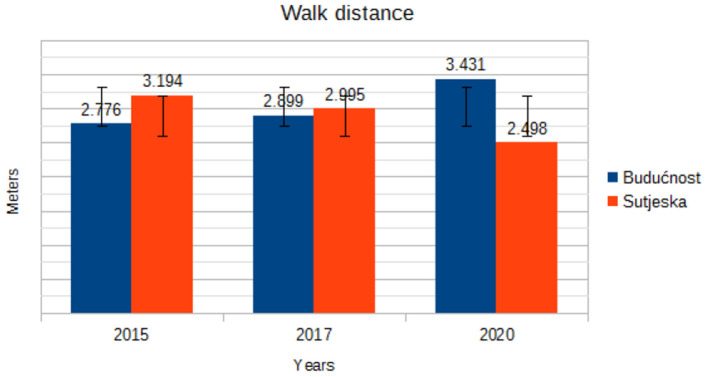
Trend in mean walk distance by years.

**Figure 3:**
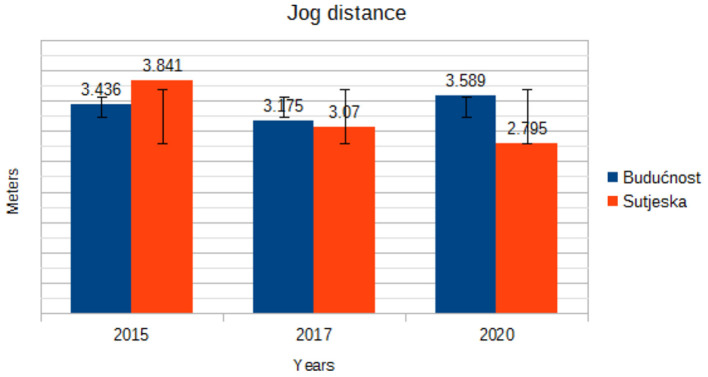
Trend in mean jog distance by years.

**Figure 4:**
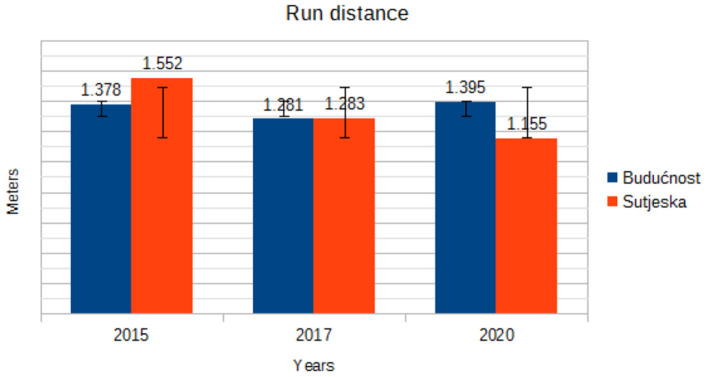
Trend in mean run distance by years.

**Figure 5:**
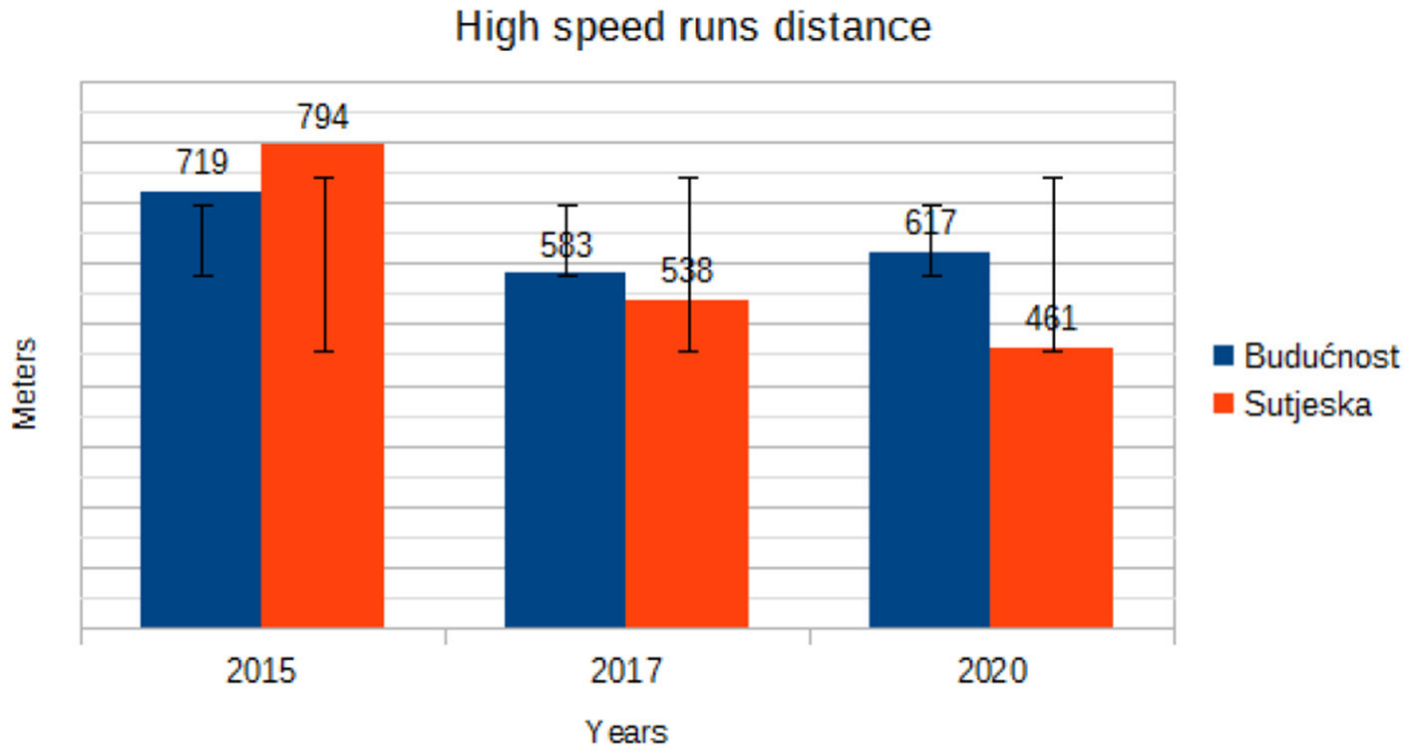
Trend in mean high speed runs distance by years.

**Figure 6:**
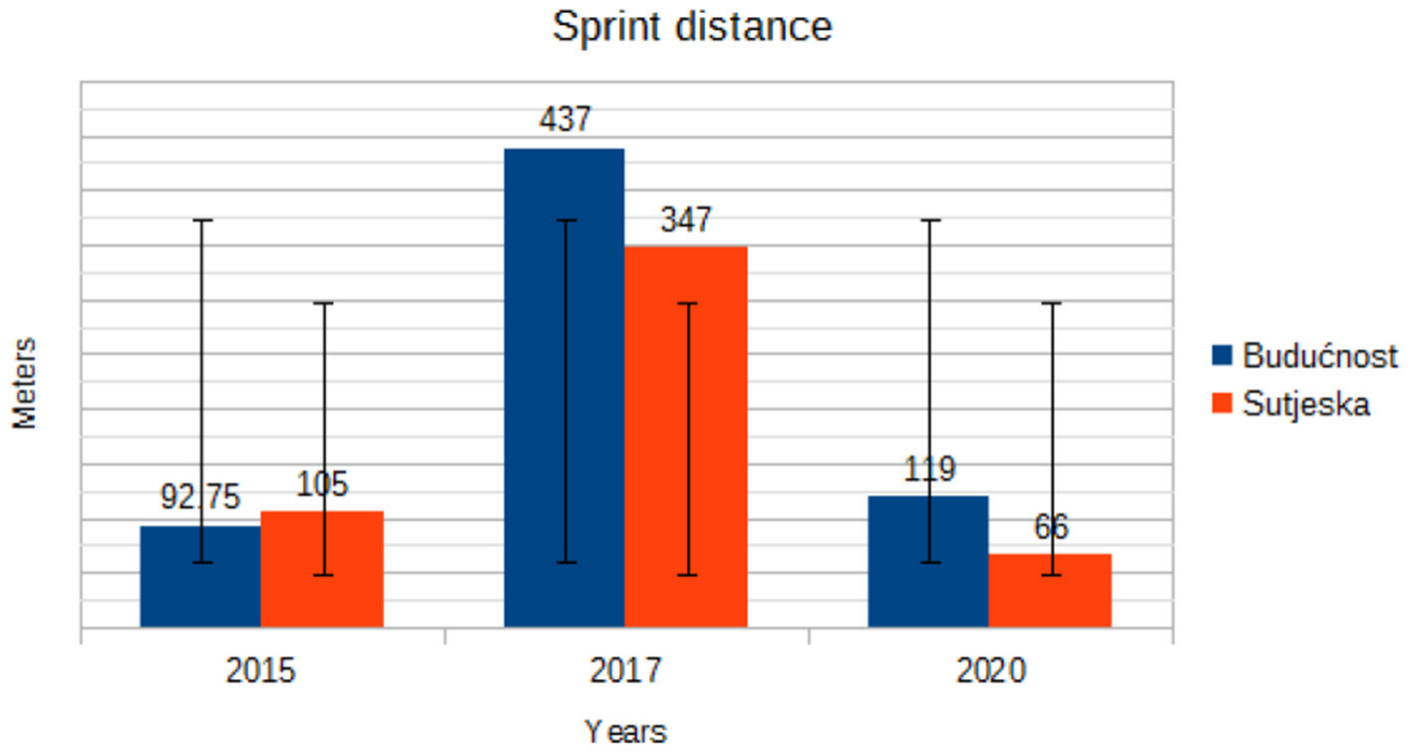
Trend in mean sprint distance by years.

Trends in running performance during the game by year are shown in the figures (Figures 1–6). Figure 1 shows the trend of the total length of running during the match. Unlike the soccer players of Buducnost, the soccer players of Sutjeska have a sharp drop in the total length of running in 2017.

Figure 2 shows the trend of walking in the game. The analysis of the graph shows that the number of meters spent walking during the game varies from year to year. The soccer players of Sutjeska reduced the trend of walking, while the soccer players of Buducnost increased the trend of walking during the game. Unlike Figure 2, which shows a walk during the game, Figure 3 shows the total jog distance of the course during the game. Inspecting Figure 3 shows that the soccer players of Buducnost have a continuous trend of jogging, while the soccer players of Sutjeska have a trend of declining jogging in all 3 years.

Figure 4 shows the downward trend in the running among Buducnost soccer players in all 3 years. The Sutjeska soccer players have seen a downward trend in all 3 years.

Figure 5 shows the high-speed running distance for the soccer players of Buducnost and Sutjeska. Looking at Figure 5, it is evident that the players of both clubs have a downward trend in the most important zone for success in top soccer with one characteristic that the players of Buducnost have a minimal increase in 2020 compared to 2017, while the players of Sutjeska have a declining trend throughout the analyzed period. In contrast, Figure 6, which provides an insight into sprint distance, shows an increase in the number of sprints at both clubs in 2017, where the players of the Buducnost made a larger number of sprints, while in 2020 there is a decline and return to identical values as in 2015.

## Discussion

The practical value of such analyses is that well-chosen performance indicators can help coaches identify the good and bad performance of individuals or teams. In this regard, match analyses help identify the physiological requirements of the sport and in examining how a particular player compares to the requirements of their event. Understanding the physiological load imposed on top players in accordance with their positional role during competitive matches (activity profile, distance traveled, intensity, energy systems, and muscles involved) is necessary when developing a sport-specific training protocol. Especially with elite athletes, the most important form of training is the one that corresponds to the use of energy and biomechanics of the planned competitive effect. Therefore, match analyses are helpful for the development of a specific training program that mimics the physiological conditions imposed by the game. Elite sports performances in soccer are a composite of the elite characteristics of physical performance, which in turn depend on several physiological characteristics, as well as on the training and health status of the individual athlete (14).

The current study aimed to analyze trends of running performance in professional soccer players in Montenegro in three competitive matches of different seasons. During the game, soccer players perform different types of movement, ranging from resting to running at maximum speed, the intensity of which can change at any time. The distance covered during the match with elite soccer players is in the range of 10,000–12,000 m (15). The results of this study indicate that the trend component for the variable total distance is on an upward trajectory for Buducnost soccer players, ranging from 8,274 m in 2015 to 9,129 m in 2020, while for Sutjeska soccer players, there is a declining trend component of 9,441 m in 2015 to 7,019 m in 2020. Di Salvo et al. (14) recorded an average distance of 11,393 m for players competing in the Spanish Premier League in the 2003/2004 season. Osgnach et al. (16) recorded an average distance of 10,950 m for soccer players competing in the Italian Serie A in the 2007/2008 season. Comparing the stated results with the current research, it is evident that the soccer players who compete in Montenegro are below the values achieved by those who compete in Europe.

In the current study, the distances covered were categorized into five levels of intensity. The trend component in the walking distance variable for Buducnost players ranges from 2,776 m in 2015 to 3,431 m in 2020, while for Sutjeska players, there is a trend component of declining walking during the game from 3,194 m in 2015 to 2,498 m in 2020. In the variables jog distance and run distance, there is a continuous trend component without large oscillations in the players of both clubs. Withers et al. (17) state that 26.3% of the total game time falls on the intensity up to 14 km/h, 64.6% on the running intensity of 14.1–19 km/h, and 18.9% on the intensity of 19.1–23> km/h. Mayhew and Wenger (18) established that a soccer player walks 46.6%, runs slowly 38%, runs quickly or sprints 11.3%, and stands without moving 2.3% of the total playing time of a game. During a match, soccer players perform different types of behavior, ranging from standing still to maximum speed runs, the intensity of which may change at any given time. However, intensity parameters are not precisely defined in these papers.

“Soccer is a non-cyclical and intermittent sport in which short-duration maximum-intensity activities, for example, sprint runs over a distance of 10–20 m, and high-intensity actions, such as counterattacks, are intertwined with activities of low and moderate intensity (marching and jogging) and with pauses, for example, standing. Sprinting is one of the most important activities in soccer, although it merely constitutes between 1 and 12% of the mean total distance covered by a player during a match, that is, from only 0.5–3% of playing time. During a competitive game, players perform 2- to 4-s long-sprint runs every 90–180 s on average. It is assumed that players of higher ability cover longer sprinting distances with higher intensity” (19). The results of our study indicate that there is a downward trend in the most important zone for success in top soccer (high-speed runs distance), with one characteristic that the players of Buducnost have a minimal increase in 2020 compared to 2017, while Sutjeska players have a noticeable declining trend throughout the analyzed period. In contrast, an increase in the number of sprints at both clubs was recorded in 2017, while in 2020, there is a decline and return to identical values as in 2015. “The amount of high-speed running is what distinguishes top-class players from those at a lower level. Computerized time-motion analysis has demonstrated that international top-class players perform 28% more high-intensity running (2.43 vs. 1.90 km) and 58% more sprinting (650 vs. 410 m) than professional players at a lower level” (20). Furthermore, Ingebrigtsen et al. (21) “found that top teams in the Danish League covered 30–40% more high-speed running distance compared to the middle and bottom teams.” In contrast, Di Salvo et al. (22) “observed that Championship players did more high-speed running and sprinting than players in the Premier League, even though the differences were small. Along the same lines, a study comparing the match performance of players in the top three competitive standards of English soccer found that players in the second (Championship) and third (League 1) categories performed more high-speed running (>19 km/h) than those in the Premier League (803, 881, and 681 m, respectively), which was also the case for sprinting (308, 360, and 248 m, respectively)” (23).

From the physiological aspect, the results of our study can be explained by the following fact: “During repetitive speed exercises, the contribution of phosphocreatine hydrolysis to the meeting of energy the demand of working muscles increases after each loading. The cool-down phase duration depends not only on the stimulation of the central nervous system but also on the rate of recovery of the autonomic nervous system functions related to the payoff of oxygen debt run up during physical exercise and on the rate of phosphocreatine resynthesis” (19).

In contrast, soccer players perform significantly less high-intensity activities when they win than when they lose or when the result is a draw. Also, if the players score a goal in the early phase of the match, they do not use the maximum of their capacities during the match. Since winning is a pleasant situation for the team, it is possible that the players have set a strategy of keeping the ball, which results in fewer sprints (24).

The limitations of this study are that only two soccer clubs from the first Telekom Montenegrin league were analyzed. Nevertheless, these two clubs are the most trophy-winning in the Montenegrin league, so they are included in the analysis. Future studies are recommended to enlarge the database. Such studies might be more suitable for detecting evolutionary trends in match-related variables.

## Conclusions

The conclusion of this study provided information on performance in Montenegrin soccer, which could consequently improve the applicability of running performance in training and competitions. Based on the obtained results, the coaches will be advised in which direction the training process should go in order to increase the performance of Montenegrin elite soccer players.

## Data Availability Statement

The original contributions presented in the study are included in the article/supplementary material, further inquiries can be directed to the corresponding author.

## Ethics Statement

Ethical review and approval was not required for the study of human participants in accordance with the local legislation and institutional requirements. Written informed consent was obtained from the participants.

## Author contributions

MJ formulated the research goals and aims, developed and designed the methodology, prepared the published work, and specifically wrote the initial draft. KG, JP, RH, and MJ prepared the published work, specifically with critical reviews, editing, and revisions. All authors commented on the draft and contributed to the final version, approved the publication of the manuscript, and agreed to be accountable for all aspects of the work.

## Conflict of interest

The authors declare that the research was conducted in the absence of any commercial or financial relationships that could be construed as a potential conflict of interest.

## Publisher's note

All claims expressed in this article are solely those of the authors and do not necessarily represent those of their affiliated organizations, or those of the publisher, the editors and the reviewers. Any product that may be evaluated in this article, or claim that may be made by its manufacturer, is not guaranteed or endorsed by the publisher.
